# Aptamer based immunotherapy: a potential solid tumor therapeutic

**DOI:** 10.3389/fimmu.2025.1536569

**Published:** 2025-02-17

**Authors:** Sarmilah Mathavan, Yew Joon Tam, Khairul Mohd Fadzli Mustaffa, Gee Jun Tye

**Affiliations:** ^1^ Institute for Research in Molecular Medicine (INFORMM), Universiti Sains Malaysia (USM), Minden, Pulau Pinang, Malaysia; ^2^ Biogenes Technologies Sdn Bhd, Jalan Maklumat, Universiti Putra Malaysia, Serdang, Malaysia; ^3^ Institute for Research in Molecular Medicine (INFORMM), Universiti Sains Malaysia, Kubang Kerian, Malaysia; ^4^ Malaysian Institute of Pharmaceuticals and Nutraceuticals, National Institutes of Biotechnology Malaysia, Gelugor, Pulau Pinang, Malaysia

**Keywords:** aptamer, tumor, regulatory, chemical modification, clinical trials, immunotherapy

## Abstract

Aptamer-based immunotherapy can be a new hope for treating solid tumors with personalized and specific approaches toward cancer therapies. Aptamers are small synthetic single-stranded nucleic acids that may bring in a paradigm shift in treating solid tumors. These are highly selective drugs applied in cellular immunotherapy, cytokine modulation, and immune checkpoint suppression. This review provides an overview of the recent advances in aptamer-based technologies with specific key clinical trials involving AON-D21 and AM003. Aptamers are potently active in immune regulation and tumor targeting. However, aptamer stability and bioavailability are seriously compromised by the issues relating to renal clearance and rapid degradation through nucleases. The latter are reviewed here along with novel improvements, some of which involve chemical modifications that greatly enhance stability and prolong the circulation time; exemplary such modifications are PEGylation, cholesterol conjugation, and the synthesis of circular nucleic acids. The regulatory aspect is also crucial. For example, in addition to specific strategies to prevent drug-drug interactions (DDIs) in cancer remediation medications, this paper underscores the need of risk assessment, particularly because of immunogenicity and organ failure. The use of aptamers is expanded by the development of SOMAmers, X-aptamers, and bioinformatics. To make aptamer-based drugs a major part of cancer treatment, future research should concentrate more on resolving existing issues and expanding their beneficial uses.

## 1 Introduction

Carcinogenesis encompasses not just genetic events but also heritable modifications in gene expression that occur without alterations to the DNA sequence, resulting in the transformation of normal cells into malignant ones. Cancer is characterized by these aberrant genetic and epigenetic alterations (1). Immune cells such as T and B lymphocytes, mast cells, natural killer cells, dendritic cells (DCs), polymorphonuclear cells, macrophages, and non-immune cells including endothelial and stromal cells coexist alongside tumor cells. They develop complex relationships with these cells, which are crucial in determining the progression and features of the tumor (2).

Cancer may have originated in the times when unicellular organisms developed into multi-cellular organisms. So far, the oldest evidence of cancer has been that in a fossil record of a shell-less stem turtle, Pappochelys rosinae, from 240 million years ago showing osteosarcoma. Until recently, tumor surgery, cytotoxic chemotherapy drugs, and radiation therapy remained the cornerstones in cancer treatment. Only from the beginning of the twenty-first century did cancer treatment with immunotherapy emerge. Inspired by the case of a soldier with a cancerous tumor regressing in an infection with Streptococcus bacteria, Coley proposed in the late 19th century that cancer could be treated through activated immune response (3).

William Coley, a bone surgeon in New York, invented “Coley’s Toxins” to treat osteosarcoma patients. His first patient, a young woman with hand osteosarcoma, died from metastatic illness after surgery. Coley’s experience inspired him to study hospital medical records for 90 sarcoma patients, focusing on one patient’s illness progression. His work is considered the “Father of Immunotherapy”. Coley discovered a case report of a sarcoma patient whose tumor shrunk after contracting erysipelas, a skin infection. He wondered if it was possible to cause erysipelas in cancer patients. German physician Friedrich Fehleisen isolated the bacteria responsible for erysipelas as Streptococcus in 1883. Coley tested his hypothesis and began administering injections of the bacteria to patients with sarcoma and finally settled on heat-killed Streptococcus pyogenes and Serratia marcescens. Coley, a pioneer in the study of cancer, injected over 1000 cancer patients and published over 150 articles. His toxins were later reevaluated in a controlled experiment, showing anticancer effects. Ehrlich believed that cancer incidence is rare, but abnormal cell production is frequent, suggesting a host defense system against cancer. Although experimental proof was unavailable, Coley’s work remains significant. Burnet and Thomas expanded on these concepts more than 50 years later, coining the term “immune surveillance” theory (3).

The idea of immunotherapy is a revolutionary approach that uses the body’s immune system to target and destroy cancer cells was first postulated by Paul Ehrlich in 1909 (4), offering a more targeted and long-lasting response compared to conventional treatments (3). This strategy combines cytokine therapies, cancer vaccines, adoptive cell therapy (CAR-T cells), and immune checkpoint inhibitors (5). As the field develops, its influence grows alongside established cancer treatments and new breakthroughs.

Multidisciplinary cancer treatment is being revolutionized by cancer immunotherapy, which is also opening up new therapeutic avenues. It continues to be included into multidisciplinary cancer care because, in certain cases, it can provide more patients long-lasting disease management (6). For many years, the foundations of cancer treatment have been radiation, chemotherapy, surgery, or a combination of these. Most patients with advanced solid tumors are not candidates for surgical removal, even if it may be curative in some circumstances (7). These treatments lack of specificity results in a high rate of recurrence and significant toxicity (8).

Additionally, the physical makeup of the tumor environment, including increased stiffness, high interstitial fluid pressure (IFP), and solid stress, poses significant challenges to delivering cancer drugs effectively—especially in dense tumors (9, 10). These forces compress blood vessels and block drugs from reaching the tumor core. High IFP limits drug movement, while solid stress from proteins like collagen further restricts diffusion (11). Combining treatments that address these physical barriers with standard therapies is showing promise in boosting drug delivery and improving outcomes for resistant tumors (12). Hence, innovative delivery methods should be explored to optimize therapeutic concentration in solid tumors and to overcome the delivery obstacles created by physical forces within the tumor microenvironment (2, 13).

Aptamer technology was developed in the 1990s, simultaneously, by two independent groups, Tuerk and Gold (14) and Ellington and Szostak (15). Aptamers known as “chemical antibodies” are a class of small single-stranded DNA or RNA oligonucleotides, with a three-dimensional structure enabling the target binding and specificity such as proteins, cells, or small molecules (16). Aptamer-based immunotherapy is a new and extremely promising method for immune regulation and cancer treatment. Aptamers have the ability to directly inhibit tumor cells, therefore serving as therapeutic agents. They may also be used in targeted drug delivery systems, which reduce toxicity to healthy tissues by directing medications precisely to tumor cells (17).

This article examines the innovative application of aptamers in cancer immunotherapy, with a focus on solid tumors. We describe synthesized single-stranded nucleic acids called aptamers and the ways in which bioinformatics techniques are advancing their development. The difficulties in using oligonucleotides to target solid tumors successfully are also covered, including problems with stability and intracellular delivery. In addition, we talked about FDA regulations for oligonucleotide-based products and the current clinical trials using the AON-D21 and AM003 aptamers.

## 2 Approaches in aptamer based solid tumor treatment

**Figure 1:**
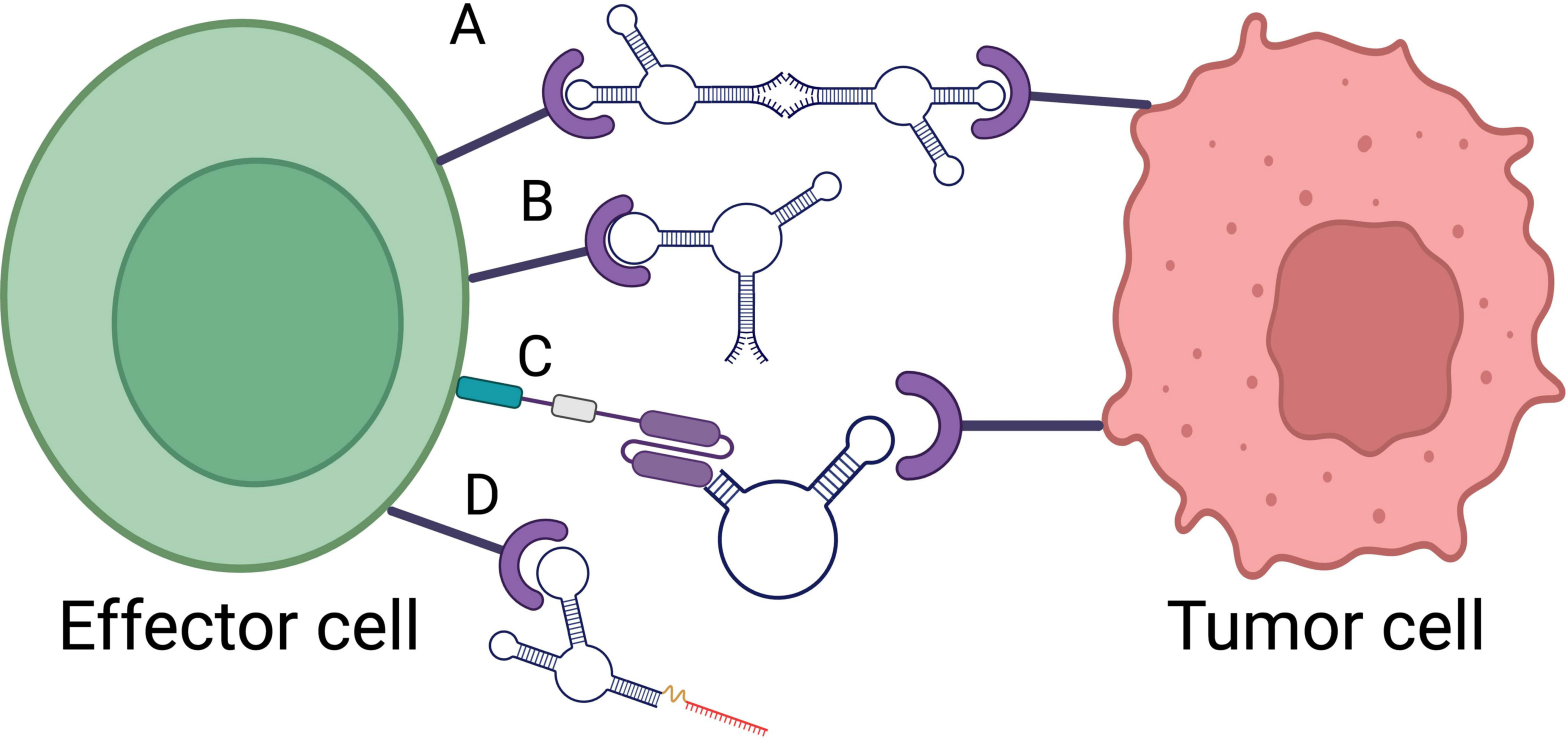
Aptamer based immunotherapy categories. **(A)** Bispecific aptamer. **(B)** Immune checkpoint inhibitor. **(C)** Car-ap. **(D)** Aptamer conjugated siRNA. Created with BioRender.com.

Aptamer-based immunotherapy can be categorized into several key approaches, each leveraging the unique properties of aptamers to engage the immune system and target cancer cells making aptamers a promising tool in cancer immunotherapy. These categories include immune checkpoint inhibition, cytokine modulation, cellular immunotherapy and bispecific aptamer (**Figure 1**).

### 2.1 Immune checkpoint inhibitors

Immune checkpoint inhibitors block the proteins needed by cancer cells, such as CTLA-4, to avoid immune destruction. It aims to restore the immune system’s ability to detect and eliminate tumour cells. In this regard, a new DNA aptamer, CTLA-4 aptamer (aptCTLA-4), preferentially increased T cell proliferation while greatly inhibiting tumor development. AptCTLA-4 acts against the CTLA-4 protein, which is a key negative regulator of T cell activation. With a high affinity attachment to CTLA-4, a dissociation constant of 11.84 nM was reported. AptCTLA-4 blocks CTLA-4 from binding with its ligands, B7-1 (CD80) and B7-2 (CD86). This interaction is important because it inhibits T cell activation since CTLA-4 and CD28 compete for binding with B7 proteins, explained by Hossen in 2023 (18). Inhibition of CTLA-4 increases the lymphocyte proliferation rate, enhancing T cell activation and proliferation (19). Aptamers are less immunogenic than monoclonal antibodies, reducing the adverse immune-related events associated with them (20, 21).

A study compares Ipilimumab, an anti-CTLA-4 mAb with CTLA-4 aptamer, highlighting both advantages and disadvantages. AptCTLA-4 increases cytotoxicity T lymphocytes and tumor infiltrating cells, preventing tumor development *in vivo* while maintaining mice’s body weight. The half-life (22) of AptCTLA-4 is less than the antibodies. However, due to lack of clinical investigation, concerns about safety and effectiveness exist. Patients with these mAbs show long-lasting clinical improvements and sometimes extended survival (23, 24), but are linked to severe immune-related adverse effect including skin lesion, colitis, endocrinopathies (24) and systemic toxicity indicated by noticeable weight loss in mice (23).

The Programmed Death-1 inhibitory receptor and its ligand (PD-1/PD-L1) are essential for immunological suppression. Tasuku Honjo’s team at Kyoto University discovered a lymphoid cell surface protein called programmed cell death protein 1, which they believe triggers apoptosis. They identified PD-1 as an immunological receptor that inhibits or negatively regulated adaptive immune responses. In an artificial activation model, the interaction of PD-1/PD-L1 inhibited T-cell proliferation and cytokine production, indicating an internal inhibitory mechanism for autoreactive lymphocyte activation. Tumor cells that express PD-L1 on their surface hamper CD8+ T lymphocytes’ cytolytic effector activities. This also showed that this inhibition might be a potential way to preventing tumor cell escape (25). The FDA approved nivolumab, pembrolizumab, and atezolizumab in 2014 and 2016, respectively for treating metastatic melanoma, urothelial carcinoma, and non-small cell lung cancer, respectively, using humanized PD-1 mAbs (26).

Antibody can inhibit the interaction by reversing tumor immune evasion and produce strong antitumor reactions. However, dermatological toxicities, diarrhea, colitis, endocrine toxicities, hepatic toxicities, pneumonitis are general adverse events (26). However, a team has identified a DNA aptamer, MP7, that specifically binds to murine PD-1 receptor’s extracellular domain with high affinity. Functional assays demonstrated that MP7 effectively inhibits PD-L1 mediated suppression of IL2 secretion in primary T cells, thereby restoring T-cell function. To enhance the aptamer’s *in vivo* stability and circulation time, MP7 was conjugated with a 40kDa PEG moiety. This PEG-MP7 retained its ability to block the interaction. *In vivo* experiments using murine colon carcinoma model revealed the treatment with PEG-MP7 significantly suppressed tumor growth. Importantly, the study reported that the anti-PD1 aptamer did not induce off target effects, such as TLR9-triggered cytokine release (27).

Furthermore, the XA library constructed by Yang and associates consists of approximately 2×10^9^ beads, each containing roughly 3×10^3^ copies of a distinct chemically modified DNA strand. XA candidates are combinations of natural and modified nucleotides such as phosphorodithioate and several modified forms of dU. Such a broad scope of chemicals enhances interactions with targets to increase binding selectivity. The library was used to find two DNA aptamers, XA-PD1-78 and XA-PDL1-82, that bind with human PD-1 and PD-L1 proteins respectively. With cell lines of overexpression, the binding affinity and specificity were verified after aptamers with proper modification were produced. The binding intensities of these aptamers were equal to PD-1 and PD-L1 antibodies. These XAs are a synthetic substitute for antibodies not only for research but also for therapeutics (28).

### 2.2 Cytokine modulation

Effective cytokine level manipulation during cancer therapies may be possible with aptamers. Targeting CD25, Axin-1 siRNAs complexed with the 4-1BB-binding oligonucleotide aptamer. CD8+ T cells that have been activated express the receptor 4-1BB. In activated CD8+ T cells, the 4-1BB aptamer-CD25 siRNA combination effectively reduced CD25 mRNA and protein levels *in vitro*. Treatment enhanced the antitumor response in mice models, both with a cellular vaccine and local radiation therapy (29) by promoting the development of long-lasting memory CD8+ T cells (30). Aptamers can deliver cytokines directly to the tumor site, enhancing immune cell recruitment and activation in the tumor microenvironment without causing systemic toxicity highlighting the importance of localized delivery in regulating aptamer-cytokine interactions (31). Downregulation and upregulation are a part of controlled modulation of cytokine levels. IL-2 upregulation at the site of 4-1BB expressing CD8+ T cells amplify immune activation while CD25 downregulation suppress immunosuppressive pathways (29), rebalancing the immune response toward anti-tumor activity.

Such fine tuning is critical in clinical applications to achieve the best therapeutic results minimizing the risk of cytokine release syndrome (CRS) and associated adverse effects (32). Since elevated blood levels are associated with increased toxicities, reducing plasma exposure while preserving efficacy has been the primary goal for the clinical development of cytokine treatments (33). Therefore, it is expected that successful therapeutic cytokine treatments need information of each patient’s unique immune profile and the ability to track changes related to the cytokine therapy (34). Dose titration and controlled-release formulations to regulate cytokine levels and minimize systemic exposure such as microsphere encapsulating cytokines (35).

### 2.3 CAR-aptamer

Aptamers can be used to enhance the specificity of T-cell therapies. CAR-ap stands for Chimeric Antigen Receptor-specific binding Aptamer developed to overcome tumor immune escape mechanisms. Designed to specifically bind to CAR-positive cells, particularly those expressing CAR19-T. It allows for traceless enrichment of CAR-positive cells without the use of traditional methods that may leave markers. It also enables accurate *in vivo* monitoring of CAR-T cell expansion, providing insights into treatment efficacy. CAR-ap-enriched CAR19-T cells demonstrate comparable antitumor activity to those enriched using antibodies (36). Aptamer-guided cellular immunotherapies can be customized based on the tumor’s unique antigen profile, improving the efficacy and safety of treatment.

### 2.4 Bispecific aptamer

The development of a bi-specific aptamer (Bi-apt) platform for cancer therapeutics is formed by two monomeric aptamers, which can specifically bind to two kinds of targets simultaneously with high affinity (37). In the context of cancer therapeutics, both the bispecific aptamers can modulate the interaction between immune effector cells and tumor cells, and promote immune cell activation and tumor cell lysis via recruiting more lymphocytes around tumor cells (38). A highly stable CD16/PDL1 bi-specific aptamer was introduced by Zheng in 2022 (39) that may directly draw in CD16-positive natural killer (NK) cells to interact with tumor cells that express PD-L1, thereby mediating a strong antitumor immunity. CP-bi-apt also can be used as an immune checkpoint inhibitor to block up-regulated PD-L1, and thus, the function of NK cells can be restored, which promotes robust antitumor immune responses. By physically bringing immune cells in proximity to tumor cells, bispecific aptamers enhance the likelihood of immune cell-mediated killing of cancer cells.

### 2.5 Aptamers in combination therapy

Aptamers’ versatility allows for their integration into combination therapies, enhancing their therapeutic potential (40). These therapies can prevent tumor development and metastasis, reduce systemic toxicities (41), and lead to innovative cancer treatment strategies like aptamer-conjugated drug delivery systems, aptamer radiotherapy synergy, and aptamer-mediated immune cell recruitment with immunotherapy.

Aptamers can be used as targeting agents to deliver chemotherapeutic drugs, siRNAs, or nanocarriers directly to tumor cells, reducing off-target effects and systemic toxicity (42). A study found a novel HER2 aptamer (HB5) through SELEX and used it as a ligand to supply doxorubicin (Dox) to breast cancer cells *in vitro*. Apt-Dox, an aptamer-doxorubicin combination, decreased its toxic effects against HER2-negative breast cancer cells while maintaining Dox’s cytotoxicity against HER2-positive cells (43). Wang presented a novel approach for automated and modular production of next-generation ApDCs for targeted drug delivery applications using solid-phase synthesis techniques. This method allows multiple drug moieties to be conjugated onto a single aptamer, resulting in high drug-loading capacity and ease of use. Similar concept with different drug called mertansine (DM1) also been carried out by (44).

Radiation therapy (RT) aiding over 50% of cancer patients every year is one of the most popular therapies for solid tumors. It gives tumor tissue the highest possible radiation dosage (45). In one study, gold nanoparticles conjugated with the AS1411 aptamer was used to test the radiosensitization effect of 4MeV electron radiation on cancer cells. Clonogenic test and Au cell uptake data combined indicated that the aptamer has increased radiation-induced destruction of cells through Au absorption. Cancer cells become more sensitive to 4 MeV electron beams as a result only with AS1411/GNPs and not with GNP alone (46). Similar study conducted by Kardani to reduce mir-155 in breast cancer, they employed complex of Au nanoparticle AS1411 aptamer antagomir 155 (47).

## 3 Structure and properties of aptamer

Aptamers’ unique three-dimensional structure, achieved through intramolecular contacts, provides thermal stability and flexibility, essential for aptamer-target interaction (48). Aptamer were suitable for large scale preparation and easily modified. Notably, aptamers lack overt immunogenicity and exhibit favorable tissue permeability and safety *in vivo* (8). Aptamer made up of 12-30kda which is 15 times smaller than antibody (49).

Aptamers adopt a wide variety of structural motifs, such as stems, hairpins, bulges, pseudoknots, and G-quadruplexes (50). Nucleic acid, hydrogen bonds, hydrophobic interactions, and van der Waals interactions play crucial roles in stabilizing the aptamer’s three-dimensional structure and ensuring selective binding to targets (51). Aromatic stacking (π-π interactions) and electrostatic forces are also integral to the binding mechanism (21). Aptamers’ binding affinities toward target proteins can be tuned by modifying their structural makeup (52). There are favored amino acid-base hydrogen bonds. Arginine and lysine strongly favor guanine base while asparagine and glutamine prefer adenine. Arginine also makes a larger interaction with thymine and adenine (53). Hydrogen bonds facilitate the majority of aptamer-target binding (54). Phosphate groups are crucial for the creation of hydrogen bonds alongside nucleic acid bases. Side chains of a protein function as hydrogen bond contributors, while the phosphate groups in aptamers typically receive hydrogen bonds (55, 56). Aptamers, being composed of nucleic acids, have negatively charged phosphate backbones. This charge property enables them to interact with positively charged target proteins, facilitating electrostatic interactions (57). This electrostatic attraction often draws aptamers to positively charged protein regions, stabilizing the binding and contributing to the specificity and high affinity of the aptamer-protein interaction​ (58).

Aptamer-protein interactions may also be influenced by the hydrophobic interaction of aromatic rings with the aliphatic side chains of proteins (59). Furthermore, it has been verified that aromatic rings take part in π−π stacking, mostly because of the π orbitals’ overlap (60). The last and most significant is the van der Waals force, a crucial binding interaction, arises from the mutual attraction of dipoles and induced dipoles (61), with its strength significantly influenced by the surface dimensions (60). Aptamer-protein interactions are well known for their high sensitivity and specificity in detecting biomolecules, even with minimal changes in structure and content. This ability is attributed to aptamers’ conformational flexibility and their capacity to undergo structural changes upon binding to target molecules, allowing for precise and selective recognition (58).

Aptamers offer superior technology over antibodies. Unlike antibodies, which usually need to be stored at −80°C to avoid permanently denature and aggregate at higher temperatures (62), they can firstly renature after heat denaturation and remain stable at room temperature for years (63). Aptamers, smaller than antibodies, minimal toxicity, and scalability of production, overcome physiological barriers like blood-brain barriers, enhancing therapeutic treatments and gaining access to underdeveloped nations, making them useful for biodefense and accessing underdeveloped nations (64) The pros and cons of antibody and aptamer are tabulated in **Table 1**.

**Table 1:** Comparison of an antibody and an aptamer.

Characteristics	Antibody	Aptamer
Material	Protein	Nucleic acid
Target	A limited target	Wide range
Size	~150kda	~20kda
Immunogenicity	High	Little to no
Development period	Few months	Few weeks
Manufacturing	Biological manufacturing	Chemical synthesis
Storage	Cold temperature	Room temperature (DNA), cold temperature (RNA)
Secondary structure	β sheets	Hairpin, loop, G-quadraplex
Batches variation	High	Low
Stability	Sensitive to temperature and pH changes	Very stable
Nuclease degradation	Resistant	Sensitive
*In vivo* half-life	Long (~a month)	Short (~20 min)
Cost	Higher	Lower

## 4 Bioinformatics application for *in silico* aptamer design

### 4.1 Conventional SELEX

**Figure 2:**
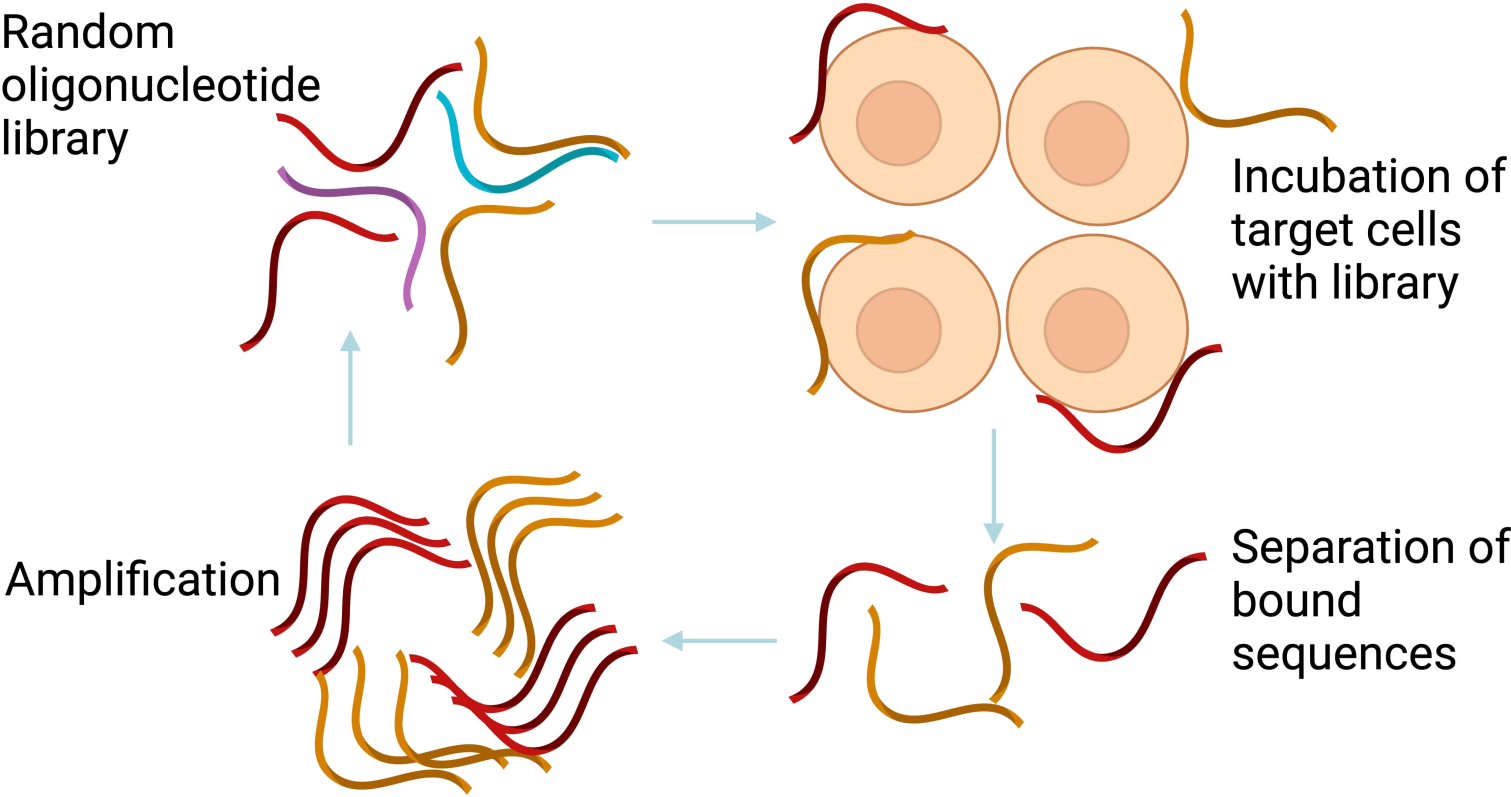
The conventional SELEX process. The conventional SELEX involves repeated rounds of selection and amplification to isolate high-affinity aptamers. Created with BioRender.com.

Aptamer was isolated usually using the conventional Systematic Evolution of Ligands by EXponential enrichment (SELEX) method, containing synthetic random nucleotide sequences length between 20-100 mers, and conserve regions at 5’ and 3’ oligonucleotides which serving as primers site for PCR amplification. The library typically contains 10^15^ – 10^24^ distinct molecules, providing a wide range of potential configurations, allowing researchers to select aptamers with the highest affinity for a ligand. SELEX generates aptamer-protein complexes by combining the library and target. The process involves removing non-specific sequences and eluding potential aptamers followed by PCR amplification. Double-stranded PCR amplicons are then separated for single stranded DNA preparation for continuation of SELEX round. SELEX undergoes multiple selection rounds to nature specific aptamer sequence and it normally took around 10–15 cycles shown in **Figure 2** (65). Enrichment of SELEX cycle was observed and selected enriched cycle was then cloned and sequence in order to identify specific sequence candidates.

Aptamers for DNA and RNA are produced differently. The recovery step involves the use of conventional PCR for DNA aptamers, which are more durable and cost-effective. In contrast, RT-PCR is required for RNA aptamers, which display stronger intra-strand interactions and varied three-dimensional structures, enhancing affinity and specificity (66).

Conventional SELEX is a tedious and lengthy process, it needs very highly trained personnel. It has been reported to have a poor success rate to isolate very specific aptamer (67), leading to the development of *in silico* SELEX, a computational-based selection that can identify high affinity aptamer, shorten the aptamer selection process within an hour and provide additional information that can be retrieved such aptamer-protein binding site.

### 4.2 *In silico* SELEX

**Figure 3:**
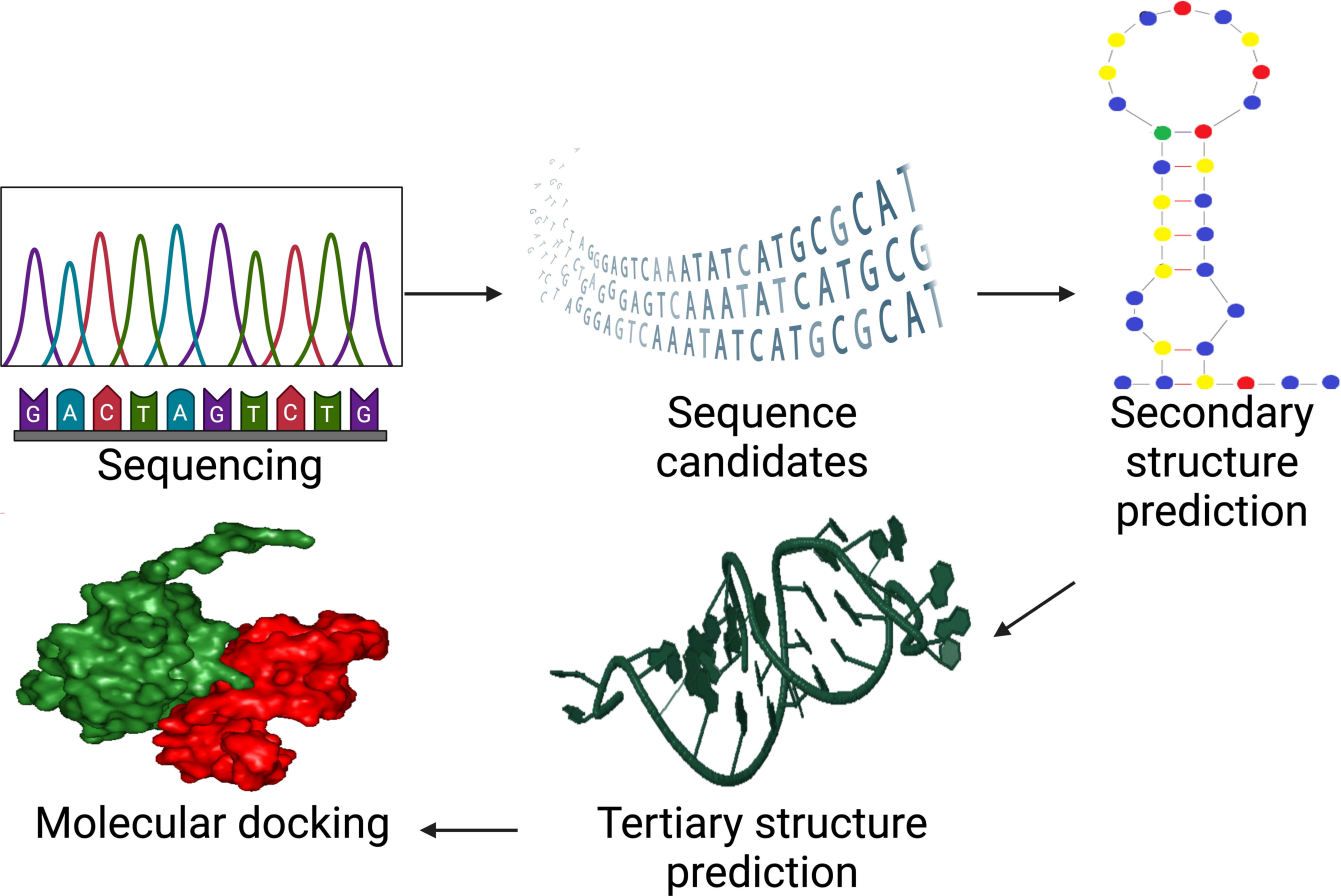
*In silico* SELEX process for aptamer selection whereas after experimental SELEX at amplification step, it incorporates sequencing, structure prediction, and molecular docking to accelerate aptamer design and optimization. Created with BioRender.com.

Chushak & Stone created the first *in silico* SELEX study by creating an extensive approach for selecting RNA aptamers (68). In order to choose aptamers with binding affinities to desired targets, the approach entails selecting RNA sequences based on their secondary structure, creating a library of 3D structures, and virtually screening the candidates shown in **Figure 3**. This technology can expedite the experimental procedures and ultimate aptamer selection by reducing the *in vitro* SELEX by 4–5 orders of magnitude (65).

In addition, since *in-silico* SELEX doesn’t require the purchase of chemicals, lab supplies, or technical staff, it is less expensive than *in vitro* SELEX. These technologies improve library design by enhancing internal random sequence length, randomization values, chemical orientation, and primer annealing affinity in constant areas. The goal is to streamline the conventional SELEX process by reducing the need for physical experimentation (67).

### 4.3 In silico

**Figure 4:**
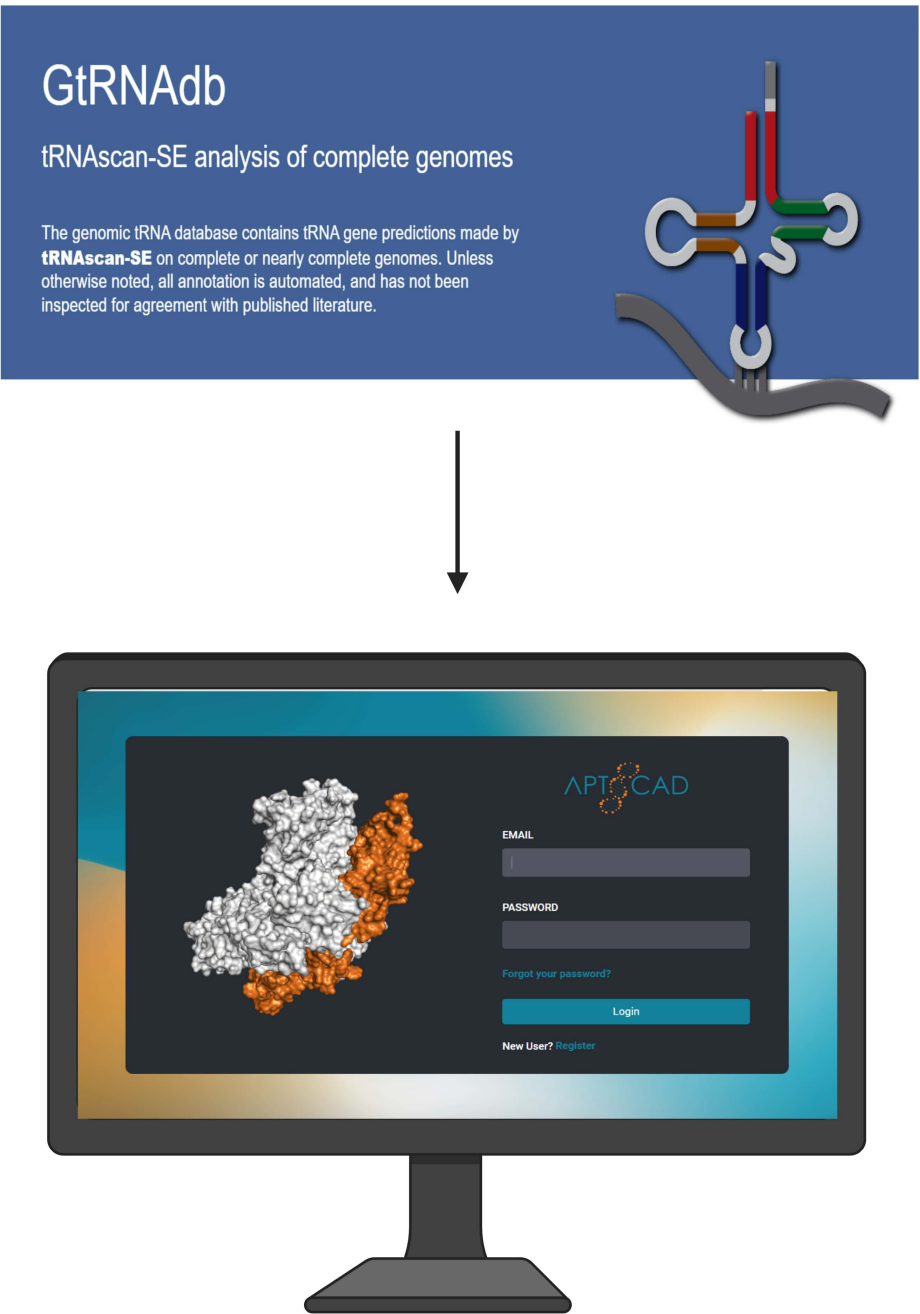
Computer interface for a software platform called APTCAD developed by Biogenes Technologies, where aptamers are designed and tested using computational tools. Natural tRNA sequences are selected from GtRNAdb, and *in silico* methods such as secondary and tertiary structure prediction, molecular docking, and dynamics simulations are employed to optimize aptamer candidates. Created with BioRender.com.

Convenient and accurate aptamer creation methods have been researched, and computer-based approaches for selecting aptamers using aptamer structure prediction have been established (69). A new modern *in silico* method was developed to find high affinity aptamers by using 3D structural modeling and natural tRNA sequences. Bio-computation can optimize these aptamers, modeling and testing them through computational docking (70) by a compilation technology introduced by Biogenes Technologies called APTCAD shown in **Figure 4**. The selected RNA sequence from https://gtrnadb.ucsc.edu/ was then truncated in relation to sequence structure involving secondary structure prediction and tertiary structure optimization, followed by structural docking simulation between target molecule and aptamer candidates.

The candidate with the least energy is chosen, and molecular dynamics simulation is used to measure stability and binding energy. The binding affinity between the aptamer and target is determined, and chemical modification or point mutation can be used to increase it (**Figure 4**) (71). The secondary structure of an aptamer is crucial in binding to a specific target substance. The prediction of the tertiary structure is significantly influenced by its secondary structure (72). The Multiple Fold (mfold) web server is a widely used bioinformatics tool for identifying secondary structures of ssDNA (73), using thermodynamic approaches like Turner’s thermodynamics table and free-energy minimization algorithms to forecast hybridization and melting temperatures (74). This online service is accessible at http://unafold.rna.albany.edu (73).

Aptamer screening aids in predicting the tertiary structures of single-stranded oligonucleotide sequences, providing molecular insights into their dynamics and structure (67). An integrated pipeline was developed to predict DNA tertiary structure using RNA-based programs, addressing the lack of available computational tools for straight modelling (75, 76). RNA Composer is a widely utilized bioinformatics tool for transforming ssDNA secondary structures into RNA tertiary models (77) at http://rnacomposer.ibch.poznan.pl/Home. The method employs a motif template-based strategy to predict intricate structures such as pseudoknotted loops and multibranched loops (78).

The conversion of RNA tertiary structures into DNA tertiary structures can be achieved by altering bases, sugar backbones, and sugar residues (76) using Discovery Studio visualizer software version 3.5 (71). Once the tertiary structures of ssDNA have been identified, molecular docking must be applied to these structures (79). A computer technology called molecular docking uses the lowest ΔG binding energy to forecast the development of an aptamer-target complex (80).

Using atomistic simulations, molecular docking also helps determine the aptamer’s binding strength against the target. Apart from interactions, aptamers’ malleability, their flexibility or rigidity, impacts their binding to the target. Stiff aptamers bind to the target’s epitope without structural changes, similar to a lock and key concept. Flexible aptamers can alter their shape to fit the target’s epitope, enhancing their binding performance (67). While there are several reliable molecular docking simulation programs available, AutoDock Vina, Glide, and AutoDock Gold have been proven to be the most effective choices with the highest ratings, while SwissDock, PatchDock, and CB Dock are other free and user-friendly resources, as shown in **Table 2**.

**Table 2:** Top options molecular docking software along with their URL address and a short description of their function.

Docking programs	URL	Function	Ref
Autodock Vina	http://vina.scripps.edu/	Molecular docking algorithm employing knowledge-based potentials and empirical scoring functions.	(81)
Glide	https://www.schrodinger.com/platform/products/glide/	Flexible energy optimization on an OPLS-AA and further refined via a Monte Carlo sampling of pose conformation.	(82)
AutoDock Gold	http://www.ccdc.cam.ac.uk/	Based on a genetic algorithm, for docking flexible ligands into protein binding sites.	(83)
SwissDock	https://www.swissdock.ch/	Based on Attracting Cavities and AutoDock Vina in providing a ranking of the ligands according to their binding free energy.	(84)
PatchDock	https://www.cs.tau.ac.il/~ppdock/PatchDock/	It facilitates rigid docking of molecules using a geometric shape. It detects complementary surfaces such grooves and protrusions between molecules.	(85)
CB Dock	http://cao.labshare.cn/cb-dock/	It uses a CurPocket algorithm to detect and rank potential binding pockets on a protein automatically before performing docking.	(86)

Conventional SELEX is experimentally driven and allows for direct, real-world validation of aptamer-target interactions, but it is time-consuming and labor-intensive. *In silico* SELEX accelerates the discovery process through computational screening, making it faster and more efficient, though it requires experimental follow-up to confirm the predicted results. DNA aptamer development using APTCAD *in silico* methods is becoming more popular as a practical way to provide a greater understanding of aptamer-target interaction.

## 5 Applications for treatment of solid tumors: clinical trials

A growing number of aptamers have entered clinical trials and are being tested as drugs of treatment for solid tumor immunotherapy (**Table 3**).

**Table 3:** Aptamers for solid tumor immunotherapy in clinical trials that are currently active.

Aptamer	Modification	Targets and application	Clinical trial ID
AON-D21	PEG-modified l-aptamer	CD88- Lung cancer	NCT05962606 Phase II
AM003	CpG Bispecific aptamer	T cell and cancer cell -Solid tumor	NCT06258330 Phase I

AON-D21 is a continuation of NOX-D19 and NOX-D20 that were previously discussed. With a 7–8 hours plasma half-life, NOX-D20 demonstrated a much longer half-life in circulation. By delaying the onset of multiorgan failure and the advancement of sepsis, NOX-D20 increases survival (87).

AON-D21 is a PEG-modified L-aptamer (Spiegelmer) that binds to C5a and C5 in both humans and mice. Without interfering with C5 cleavage, it selectively blocks C5a from binding with its receptors. AON-D21, a plasma stable anti-C5a L-aptamer, in the *in vivo* model of A549M1 cell bone metastases. C5 or C5a was utilized as a negative control as the Spiegelmer of the reverse AON-D21 sequence, RevAON-D21, is unable to bind any of them. Every other day starting the day before the A549M1 cells were injected intracardiac, AON-D21 and revAON-D21 were administered intraperitoneally (10 mg/kg) in saline. The tumor volume was calculated and measured with the use of an electronic calculator. C5aR1 activation induces the production of osteoclastogenic factors and raises the risk of bone metastases from lung cancer (88).

A DNA pattern known as the CpG motif, which is abundant in unmethylated Cytosine-phosphate Guanine (CpG) nucleotides and common in bacterial DNA, triggers an immunological response in the host. The result of Aummune’s cutting-edge customized therapeutic platform, which finds functional aptamers capable of killing tumor target cells, is AM003, a Bispecific Personalized Aptamer with a T cell engager arm and a tumor-targeting arm. After hybridization, AM003’s two ssDNA aptameric arms are intended to generate a new immunostimulant CpG pattern. Intratumoral injection of AM003 significantly changed the tumor’s immunological environment by increasing the invasion of B and CD8+ CD4+ T cells. By enhancing T cell infiltration, stimulating the innate immune system, and facilitating subsequent efficient T cell activation through the compound’s T cell engager arm, AM003 tackles the problems associated with immunological resistance (89).

## 6 Challenges of unmodified aptamer in immunotherapy application

Although aptamers hold significant promise, most have failed to achieve the required safety and efficacy benchmarks in human clinical trials. A number of factors limit their use in therapeutics, including rapid degradation through nucleases, rapid clearance via renal filtration, and a lack of chemical diversity among others (90). Aptamers consist of nucleotides, and hence they might be degraded by nucleases under most *in vivo* or *in vitro* tissue culture conditions (91). These could cause strand breaks in either one or both strands of the nucleic acid, which will render it ineffective. Nucleases are enzymes capable of cleaving the phosphodiester bonds that join nucleotides in DNA and/or RNA. While the endonucleases can cut at different points along the polynucleotide strand, either single-strand or double-strand breaks, there exist exonucleases-which cleave nucleic acids from the ends, either 5′ or 3′ (92).

Because of their relatively small size, approximately 10-20 kDa or less than 5 nm in size, aptamers eliminated from the blood very fast after *in vivo* administration. By intravenous administration, they get cleared off the blood within approximately 10 minutes (93). Renal filtration can rapidly excrete small aptamers when they are introduced into the bloodstream, even if stabilizing backbone alterations are utilized (66). This reduces their therapeutic effectiveness since they do not remain in the body long enough to perform their intended function. These challenges make their development more complex and limit their wide application. Nevertheless, this property is not necessarily a drawback. It will rely on the intended application of the aptamer. On the other hand, due to their small dimensions, aptamers show improved tissue permeability and can reach their target more efficiently before being cleared by the kidneys (94).

Aptamers, unlike antibodies assembled into amino acids, are composed of four nucleotides: guanine, cytosine, adenosine, and thymine or uracil, compared with 20 amino acids in the case of antibodies (95, 96). Aptamers, as a result of their highly minimalist chemical structure devoid of even side chains and complex functional groups, have limited structural and chemical diversity. Their ability to effectively engage some targets would therefore be diminished (91, 97). The inventive structural development of aptamers has therefore been pushed forward into potentially more viable routes for aptamer-based therapeutics by the numerous problems that have been found in their development, such as those covered above.

From a clinical translation viewpoint, the most major challenge for aptamer medicines is their stability, particularly their sensitivity to fast nuclease breakdown and renal clearance. These obstacles significantly restrict their absorption and therapeutic effectiveness, necessitating chemical changes to optimize their pharmacokinetics. Chemical modifications are strategies that can improve stability and lengthen circulation duration, but they cannot resolve this issue adequately. Despite these advances, aptamers’ half-lives remain much shorter than those of monoclonal antibodies (mAbs) (62). Among the key issues that may answer the question of whether aptamers can remain intact and functional within biological systems, stability is the cornerstone challenge for their successful clinical translation. Stability is crucial for aptamer’s functionality in biological system (98). It therefore constitutes the primary obstacle to their effective clinical translation.

There is no documented evidence of tumor resistance to aptamer-based therapies in clinical settings till date. However, tumor resistance mechanism must be considered while developing any treatment approach including aptamers (99).

## 7 Strategies to overcome challenges

Improving the stability of unmodified aptamers is critical, especially in biological environments where nucleases can degrade them quickly. Here are several strategies to enhance the stability of unmodified aptamers. Modifying the 3’ and 5’ ends shields aptamers from exonucleases, while phosphorothioate linkages enhance protection against endonucleases (100).

Resistance mechanism in tumors such as multidrug resistance (MDR) also pose a challenge for aptamer-based therapies. One study demonstrated that aptamer d3 binding was revealed to block the MDR of the tumor cells and increase the accumulation of intracellular anticancer drug, which lead to a boost to the cell killing (101). Multifunctional aptamers, which target multiple pathways can further reduce the likelihood of resistance alongside with combination therapies reducing reliance on a single mechanism (102).

Adding modifications like inverted thymidine (inverted T) at the 3’ end is the most common modification (103). The presence of the inverted dT had a modest approximately threefold effect on stability according to Kratschmer’s study. The synthesis of aptamers with 3’ inverted thymidine modification requires the use of modified CPG, where the 5’ hydroxyl group of the first nucleoside is attached, and then the chain is elongated in the typical 3’→5’ direction (104). Incorporating a 3’ inverted dT residue can also prolong duration to several hours instead of an hour for DNA’s half-lives (105). Pegaptanib includes a 40 kDa polyethylene glycol (PEG) unit at the 5’ end to aid in renal clearance, along with a 3’-3’-linked deoxythymidine residue to enhance resistance to nuclease degradation (106). Despite these modifications, pegaptanib maintained an exceptionally high affinity for its VEGF165 target and demonstrated prolonged *in vitro* stability stated by (107).

Modifying the phosphodiester backbone changes the metabolic pathway, improving nuclease-resistant stability, as seen with phosphorothioate linkages (108). In this modification, one of the non-bridging oxygen atoms in the phosphodiester bond is substituted with a sulfur atom (109). In 1967, Eckstein (108) first introduced the phosphorothioate (PS) modification strategy to improve RNA’s resistance to ribonucleases (RNase). During the oxidation step, the standard iodine solution was replaced with phenylacetyl disulfide solution (5 g in 2 ml pyridine) to achieve PS modification. Since phenylacetyl disulfide lacks stereoselectivity, the resulting oligonucleotides are racemic mixtures (108). Based on research conducted by researcher Wu, the PS modified sequence WW-24 demonstrated outstanding selective anti-melanoma activity (A375 cells, ∼25 nM, 80%), targeting both hnRNP A1 and hnRNP A2/B1, exhibiting a stronger antitumor effect and prolonged accumulation time *in vivo* (110).

A circular nucleic acid (CAN) is defined by its closed-loop structure formed through covalent bonding between the two ends of a linear nucleic acid molecule (111). Most of the circular nucleic acids are prepared through the chemical or enzymatic ligation of linear oligonucleotides (112). Chemical ligation in this respect generally refers to the use of cyanogen bromide, BrCN, or 1-ethyl-3-(3′-dimethylaminopropyl) carbodiimide for connecting DNA-RNA hybrids. However, this technique also has its drawbacks of giving low ligation efficiency, and it may have some biosafety risks. Another drawback of the technique is that it generates 2′, 5′-phosphodiester bonds instead of the natural 3′, 5′-phosphodiester bonds. One of the typical protocols for the generation of cDNA involves the use of ligase for the circularization process (113). T4 DNA ligase is one among the commonly used ligases in this process that catalyzes the ligation of the 3′-hydroxy and 5′-phosphate ends of a ssDNA molecule with the help of a splint DNA strand (112).

Additionally, the enzyme CircLigase (114) or ssDNA ligase (115) can be employed to catalyze the joining of ssDNA that has complementary ends. Recently, a nuclease-resistant circular bivalent aptamer system was created, offering enhanced stability and improved tumor-targeting abilities *in vivo* (116, 117). This system, formed by ligating two linear aptamer sequences, demonstrated increased thermal and physical stability in biological environments while showing superior tumor cell targeting (118).

To address the quick renal filtration issue, two most common methods have been reported, such as attaching cholesterol and PEG (119). More importantly, PEGylation has already been established in extending the circulation life of various therapeutics, including proteins and small molecules, which in turn drives substantial progress in biologics development and drug-loaded nanoparticles (120). PEGylation was first conceptualized as a method to overcome the problem of immunogenicity of non-human derived proteins intended for human use, and the concept was first envisioned by Frank Davis in the late 1960s. PEG was chosen because it formed the hydrophilic component of a clinically utilized block copolymer (Pluronic, made up of PEG and polypropylene glycol). Davis theorized that attaching PEG to proteins or small molecules would make them less detectable by the immune system, thereby reducing immune responses while extending their circulation and functional lifespan (121).

The PEGylation can be attributed to the increase in hydrodynamic size, which leads to a reduction in renal filtration due to PEG conjugation (122). PEG with a molecular weight below 30 kDa is removed from the body via the kidneys, above 20 kDa is excreted through the feces (123). Macugen™ (pegaptanib sodium, Pfizer) is another significant PEGylated small molecule drug or aptamer. It is the first aptamer-based therapeutic approved for human use working as a selective antagonist of vascular endothelial growth factor (VEGF) (124). The FDA has approved Avacincaptad Pegol (Izervay) from Iveric Bio/Astellas for geographic atrophy due to age-related macular degeneration. It is the second RNA aptamer and complement-targeted drug to receive FDA approval for this condition (125).

Cholesterol conjugation at 5’ end improves aptamer pharmacokinetic properties by creating a cholesteryl-oligonucleotide (cholODN) complex with low density lipoprotein (LDL). This complex exhibits exceptional resistance to nuclease hydrolysis, extending half-life (117, 126). A modified RNA aptamer inhibited Hepatitis C virus replication *in vitro*. It was later derivatized with cholesterol to form chol-aptamer, which successfully inhibited HCV RNA replication in a cell-based system. Systemic administration was well-tolerated by mice and increased plasma retention time (127). By increasing aptamer hydrophobicity, cholesterol lowers plasma clearance rate and lengthens half-life. Aptamers combined with cholesterol have better biodistribution patterns and longer circulation half-lives, according to preclinical research (119).

Gold and colleagues developed SOMAmer, a method that mimics protein side chains by attaching amide groups to deoxyuridine triphosphate at the 5′ position aggrandizing the diversity of nucleic acids (128). In their respective investigations, researchers Li (129) and Ji (130) highlighted how this alteration improves binding affinities, provides hydrogen bonding partners, and avoids linkage bond rotations. SOMAmers incorporate modified nucleotide bases to make aptamers more effective at interacting with hard-to-reach sites that classical aptamers struggle to access. One goal is to enable aptamers to interact with hydrophobic regions, which can be crucial for the activity of toxins but are challenging for traditional nucleotides to target (131). SOMAmers interact with target molecules during the SELEX process through more hydrophobic and less polar interactions, involving fewer hydrogen bonds and charge–charge interactions than standard aptamers. They demonstrate slower dissociation rates (over 30 minutes) while avoiding covalent and permanent bonds that are often linked to higher dissociation constant (Kd) values (132, 133). An added benefit of these modifications is the significantly extended half-life of the aptamers *in vivo* (133). Even though SOMAmers has extensive purification and characterization compared to standard SELEX, they exhibit stable chemical structures, great nuclease resistance, and binding properties equivalent to antibodies. Personalized SOMAmer reagents for a specific protein target may be made faster and cheaper than antibodies (134).

Similarly, a new generation of hybrid aptamers, called X-aptamers, has been developed incorporating amino-acid-like side chains or drug-like ligands (X). AM Biotechnologies, Houston, USA, developed the X-aptamer selection kit, which was used to pick X-aptamers (135). These combine monothiophosphate-backbone-substituted aptamers for enhanced stability and protein binding with chemically modified uridine (referred to as X) that allows drug-like compounds or additional functionalities to be attached to the aptamers. The substitution of one of the oxygen atoms in the phosphate group with sulfur is the modification that makes the aptamer resistant to nuclease degradation (136). Utilizing this technology, selection of X-aptamers against two immune checkpoint proteins, PD-1 and PD-L1, have been developed (28). X-Aptamers were considered to be the next generation aptamers as X-aptamer showed therapeutic potential in acute pancreatitis (137). Generally, a bead-based X-aptamer library was used to find an X-aptamer that interacts to the target precisely (135). The PCR results were combined after 25 cycles and submitted to the manufacturer for Illumina next-generation sequencing. The company synthesized potential x-aptamers with a 5’ biotin tag, requiring 1-2 weeks for the entire procedure, which includes synthesis and sequencing (136).

Furthermore, selection of the best chemical modification approach, such as PEGylation, cholesterol conjugation, or phosphorothioate linkage for aptamers, depends on tumor type, patient characteristics, and therapeutic application has been discussed in general by (138). PEGylation is the best modification for aptamer stability and half-life circulation in solid tumors with poorly vascularized regions with dense extracellular matrix that leads to slower drug uptake, such as lung, colon, and breast cancer (139). Dosing may need adjustment due to individual differences such as liver and kidney function (140).

Cholesterol modified aptamers improve cellular uptake and enhanced interaction lipid bilayer hence its very beneficial for targeting lipid-rich tumors such as ovarian, prostate and glioblastoma (140–142). Patient-specific lipid profiles should thus be considered when formulating and delivering cholesterol-conjugated aptamer treatments in order to provide clear therapeutic recommendations (143).

PS linkages enhance aptamer stability and binding affinity, making them ideal for pancreatic cancer (144), brain tumors due to their ability to cross the blood brain barrier (140), also melanoma since phosphorotioated aptamer have special anti-melanoma effect (110). However, they can also increase immunogenicity and trigger an inflammatory cytokine response, requiring cautious treatment.

## 8 Food and drug administration guidelines on oligonucleotide products

The term “drug development platform technology” describes the broad variety of tools, processes, and platforms set up with the purpose of further increasing the efficiency in development and manufacturing of pharmaceuticals. They are key drivers in accelerating the research of drugs, preclinical trials, clinical testing, and medicinal production (145). Regulatory frameworks are equally crucial in this development. Organizations such as the EMA (146) provide standardized regulations to ensure the safety, efficacy, and quality of drugs created using platform technologies, simplifying regulatory approval processes. The FDA introduced the most significant guideline in May 2024 (147), establishing criteria for the platform technology designation under Section 506K of the FD&C Act, aimed at improving drug development efficiency.

FDA recommends oligonucleotide therapeutic evaluations, including QTc interval prolongation assessment, immunogenicity risk, hepatic and renal impairment assessments, drug-drug interaction studies (148). The most important factors in assessing cardiac risk are the QT intervals, which are mediated by the flow of ions such as sodium, potassium, and calcium across receptors in the cell membrane (149). These may cause severe conditions including syncope, cardiac arrest, and sudden death especially when QTc is further extended. Normal QTc intervals normally are normally measured as < 450ms for men and < 460ms for women (150). A baseline ECG should be taken. It may be repeated once the drug reaches steady state. A baseline ECG should be taken and may be repeated once the drug reaches steady state (151).

Assessment of the risk of immunogenicity is a major component in the development of biotherapeutic drugs and is also one of the factors taken into consideration in the overall benefit-risk evaluation. This generally includes an assessment of the likelihood of inducing an immune response and any potential clinical consequences if such a response occurs (152). As the field advances with immunomodulatory multi-specific therapies, pharmacological or mechanism of action (MoA)-based immunogenic risk factors are becoming increasingly significant (153). However, the risk of an immune response can be lowered by the molecular weight of certain biotherapeutics (154), and high doses may promote long-term immune tolerance. Additionally, it is important to consider the patient’s immune status and prior exposure to relevant treatments (155).

A comprehensive immunogenicity risk evaluation includes a summary of the drug’s target, its intended use, and its structure. It considers sequence-related risks by identifying which sequences may bind to immune cells and evaluating the risks associated with the drug’s mechanism. Population-based risks are also assessed to determine if specific groups may have varying incidences of immune responses. Additionally, the evaluation addresses quality attribute-related risks, such as impurities, and includes factors like the expected half-life, dosing regimen, sampling plan, and a schedule for sharing immunogenicity data (155).

Healthy patients in the renal study needed to have a creatinine clearance of ≥90 mL/min as estimated by the Cockcroft-Gault equation. The hepatic study requires a creatinine clearance of at least 60 mL/min. The hepatic impairment trial was an open-label, phase 1 parallel-group, multicenter research designed to assess PK, safety, and tolerability in people with mild, moderate, and severe liver impairment to healthy participants. In other words, liver damage reduces intrinsic drug clearance due to decreased functional mass of liver cells and lower hepatic blood flow caused by liver fibrosis scarring (156).

In cases of severe hepatic impairment, the drug’s initial dose might need to be reduced and closely monitored. According to FDA guidelines, studies aimed at estimating the impact of renal function on drugs with a molecular weight below 69 kDa on their PK should be evaluated. Therapeutic drug monitoring (TDM) could be employed for dose adjustment (157). Myotoxicity, another potentially serious and severe side effect, may cause rhabdomyolysis, acute renal failure, disseminated intravascular coagulation, and death (158, 159).

Drug-drug interactions (DDIs) pose a serious global health threat because their adverse effects can be lethal (160), not just from the prescription drugs, but dietary and herbal supplements are also dangerous (161). One out of every 25 chances of supplements usage with prescription drugs leads to DDIs (162). While most DDIs are mild, some may be severe or life-threatening and may necessitate medical intervention. DDIs occurring with anticancer drugs may have serious consequences on both the treatment outcomes and toxicity. Evidence-based electronic surveillance for DDI is now crucial to increasing treatment efficacy while ensuring the safety of patients. In recent years, within the last ten years, several therapeutic drug managements (TDM) have been developed to manage DDIs (162). Physiological changes with age, food interactions, and other factors can alter drug absorption, distribution, metabolism, or excretion. New technologies can further enable the creation of more innovations in personalized medicine, therefore supporting the management of drug interactions (163).

A study on the first-in-human whole-body dynamic pharmacokinetics of aptamer incorporates a range of tests and evaluation to monitor and manage potential adverse effects. Preclinical toxicity evaluation is one of the crucial steps in the process where a single dose toxicity test was conducted to assess the aptamer’s safety. This involved monitoring dosage levels, clinical observation as well as changes in body weights, hematological markers, bone marrow micronucleus formation. Additional tests such as the mutagenic, carcinogenic and genotoxicity test were performed, including a reverse mutation assay and chromosomal aberration test in mammalian cells. In clinical studies, patient enrolment and clinical study design involve detailed recording of patient characteristic such as diagnosis of cancer or a medical history of cancer. Prior to treatment, a comprehensive physical examination is performed including the assessment of vital signs, routine blood tests, liver and kidney function, blood human chorionic gonadotropin (HCG) for females and electrocardiogram. These clinical assessments ensure that patients are suitable candidates for the treatment and provide baseline data for monitoring adverse effects during the study. In addition, whole-body dynamic PET imaging is employed for pharmacokinetics studies to observe biodistribution and the clearance patterns of the aptamer. This imaging provides real-time data on how the aptamer interacts with the body (164).

## 9 Future directions

The advancements in aptamer-based immunotherapy for solid tumors underscore the potential for this technology to revolutionize cancer treatment. However, future study needed especially on stability since it still remains a significant barrier. Imidazole thymine is a novel discovery that has not yet been extensively studied or validated yet, but it mediated duplex stabilization (165, 166) compared to conventional chemical alteration. Expanding the strategy of aptamer integration with immune cells like Natural Killer (NK) to enhance efficacy for more personalized targeted cancer treatment is an attractive area for future study. The surface of NK cells was used to anchor aptamers for cell-specificity. This approach has yet to be explored (167).

## 10 Conclusion

Aptamer-based immunotherapy, in short, stands at the edge of revolutionizing cancer treatment, especially treatments against solid tumors. This is considered a very promising emerging field because the aptamers have special advantages in front of the traditional therapies, such as high specificity, lower immunogenicity, and ease of modification. Aptamers can act not only as therapeutic entities per se but also as vehicles and immune system modulators in the context of different treatment modalities that range from checkpoint inhibition to cytokine modulation, cellular immunotherapy, and bispecific aptamers.

It is indicated that aptamers may have a good future in solid tumor immunotherapy, according to trials such as AON-D21 and AM003. However, various limitations arise due to aptamer instability *in vivo* and their very short half-life, which results from rapid degradation by nucleases or renal clearance. Such are surmountable with particular chemical modifications including PEGylation, cholesterol conjugation, and circular nucleic acid, which enhance the stability of aptamers, improve pharmacokinetics, and prolong the circulation time.

According to the regulatory guidelines, it is also pinpointed that development should consider immunogenicity and impairment of organs, including renal and hepatic function, which may affect drug metabolism and safety. Drug-drug interactions remain important, especially in patients with cancers undergoing treatments; thus, thorough evaluations and personalized treatment approaches are called for.

Further advances in bioinformatics-driven aptamer design, *in silico* SELEX methods, and next-generation aptamer technologies, such as SOMAmers and X-aptamers, are foreseen to result in enhanced therapeutic efficacy and broader clinical use against cancer and other diseases. Remaining challenges regarding stability, delivery, and regulatory compliance will require research emphases in the future to fully exploit the potential for aptamer-based therapies in the clinic.

Personalized medicine and improved management of drug interactions will ensure optimum treatment for tumors, especially when new therapeutic combinations become available. Further studies in aptamer-based immunotherapy should be able to overcome the present obstacles and offer new effective treatments with less side effects to patients affected by solid tumors and beyond.
